# Cytotoxic, antioxidant, and antimicrobial activities of Celery (Apium graveolens L.)

**DOI:** 10.6026/97320630017147

**Published:** 2021-01-31

**Authors:** Mohammed Saleh Al Aboody

**Affiliations:** 1 Department of Biology, College of Science, Al-Zulfi-, Majmaah University, Majmaah- 11952, Riyadh region Kingdom of Saudi Arabia

**Keywords:** Apium graveolens Linn, cytotoxic, antioxidant, antimicrobial activity

## Abstract

Celery (Apium graveolens Linn, Family: Apiaceae) is a common edible herb used as a spice in the traditional medicine of several nations since time immemorial. The whole plant is extensively used in cooking as soups and salads. A. graveolens has various pharmacological properties such as anticancer, anti-obesity, anti-hepatotoxic, and antihypertensive agents. Hence, it is of interest to document the in vitro cytotoxic, antioxidant, and antimicrobial activity of A. graveolens. The plants were collected in the local market, shade dried, and different parts of the plants were extracted with 70% ethanol using a cold maceration process. Antioxidant tests were performed based on the various radical scavenging methods. Antimicrobial activity and MIC were completed using the respective cup-plate and two-fold serial dilution method. In vitro cytotoxic studies were achieved by the MTT; Sulphorhodamine B assayed total cell protein content. DLA and ESC cells determined the short-term toxicity. The leaf extract exhibited significant antioxidant properties against NO, DPPH, ABTS, LPO, and HPO methods. Thus, potential inhibition against Gram-positive, Gram-negative, and fungal strains within the MIC ranges of 250-500 µg/ml was observed. All the extracts of the plant presented in the study revealed greater cytotoxicity effects against five respective cancer cell lines, L6, Vero, BRL 3A, A-549, L929, and L-929 with the ranging of 443-168.5 µg/ml. Thus, we show that A. graveolens possess a potential cytotoxic, antioxidant, and antimicrobial activity.

## Background:

Medicinal plants are used worldwide due to its antioxidant and antimicrobial effects that become most popular due to the growing ratio of drug-resistant microorganisms [1]. Nevertheless, abuse of antibiotics has become the primary cause of the development and spreading of multidrug-resistant strains of various classes of pathogens [2-5]. The antimicrobial effects of diverse medicinal plants and there by products have been extensively studied and several clinically important compounds have been validated [6,7]. The plant extracts and their derivatives have been practiced for hundreds of years in folk and alternative medicine, food preservation, pharmaceuticals, and natural remedies [8,9]. Some vegetables, fruits, spices, herbs, and various parts of the plant extracts have been described to be potential antimicrobial, anticancer, anti-inflammatory, anti-aging, and antioxidant properties [10-12]. These antimicrobial/antioxidant properties are mainly based on the occurrence of major bioactive compounds, including alkaloids, phenolic acids, terpenes, glycosides, and flavonoids [13,14]. Even different vegetables have also known to be antimicrobial, anti-inflammatory, anti-aging, anticancer, and antioxidant properties [15,16]. Earlier studies have also demonstrated that the antibacterial and antifungal effects of different plants against various enteric bacteria and common fungi by using their plant extracts and derivatives [17-20]. Celery (Apium graveolens L. Family: Apiaceae) is commonly consumed as a vegetable and flavoring ingredient in cooking in various nations of Asian Countries. The whole plants including, leaf, stem, root, and seed are extensively used in cooking as soups and salads. It is most popular based on its unique aroma and essential oil. The plant is a good source of carotenes, tocopherols, and vitamins with high quantities of secondary metabolites including ﬂavonoids, alkaloids, terpenoids, and phenolic acids [21,22]. Indeed, the plants are commonly practiced in folk medicine to heal numerous ailments including, asthma, bronchitis, hypertension, diabetes, gastrointestinal disorders, urinary calculi, visceral spasm, impotency, and hepatitis [21, 23]. The seed of A. graveolens contains essential oil with a distinctive aroma and contains various coumarins rich compounds [24,25]. Celery has various pharmacological activities, including, hepatoprotective [26], cholesterol-lowering [27], antioxidants [28,29], anticancer [30], anti-inflammatory [31], analgesic [32], cardioprotective [33], anti-infertility [34], larvicidal, and mosquito repellent activity [35]. Earlier studies reported the antioxidant and antimicrobial activity of celery in Pakistan, South Korea, and the U.S.A. [28,29, 36]. However, antioxidant and antimicrobial and cytotoxicity investigation has not been yet performed using celery in Saudi Arabia. Phytochemical contents of the plant are often varied within the species as well as nationwide. It is regulated by numerous extrinsic factors including, latitude, longitude, rainfall, physicochemical parameters of water, atmospheric temperature, moisture, soil content, and photoperiods [37]. Due to the adaptation of hydric or salt stress, plants enable to increase their osmotic tension and produce various phytochemicals including, essential oils, organic acids, alkaloids, and saponins that attract insects as well as prevent predators [38]. Thus, the same species in the plants can alter their phytochemical composition due to their extrinsic factors. A recent development toward emerging herbal drugs as complementary to synthetic medicines has elicited greater attention due to their beneficial activities. Based on the attention, herbal plants and vegetables can be characterized as bioactive ingredients, and elucidate their mode of action, therefore, creating them feasible therapeutic ingredients. Therefore, it is of interest to document the in vitro antioxidant, cytotoxic, antibacterial, and antifungal activity of A. graveolens.

## Materials and methods:

### Plant materials and chemicals:

Apium graveolens L. was acquired from the local market. MTT, DPPH, and ABTS were obtained from Sigma-Aldrich (St. Louis, MO, USA). BSA, DMEM, RPMI 1640 medium, HBBS, trypsin, and PBS were procured from Biosera (Manila, Philippines). DMSO, Folin-Ciocalteu reagent, sodium carbonate, nutrient broth, hexane, and methanol were acquired from Merck & Co. Inc., Darmstadt, Germany. Standard antibiotics were procured from EBEWE Pharma GmbH Nfg.KG, Mondseestraße, Austria and Invitrogen, San Diego, CA, USA.

### Preparation of the plant extracts:

The whole plant of A. graveolens L. was splashed with tap water and was shadow air-dried at 37°C and was applied for the method of solvent extraction. The different parts of the plant (stem, leaf, root, whole plant) were exposed to the method of cold maceration by ethanol (70%). The suspension was exposed to evaporate and the residual crude extract was kept in the freezer for future investigation. The hydroethanolic suspension of stem, leaf, root, and the whole plant was subjected to the phytochemical screening tests as per the standard methods described earlier [39-41].

### Antioxidant Activity:

Radical scavenging method- ABTS:

The assay of ABTS•+ and DPPH has commonly used methods for the determination of the antioxidant capacity of natural products. The free radical scavenging method (ABTS) was adopted according to the earlier publications [42, 43]. ABTS (54.8 mg) was dissolved in distilled water (50 ml) to be prepared as a 2 mM solution, and added along with 0.3 ml of 17 mM potassium persulphate, and kept at 37°C overnight. Added to 0.4 ml of each concentration of the plant extracts and standards, DMSO (1.0 ml), and ABTS (0.2 ml) to make up the suspension of 1.6 ml and kept 20 minutes for incubation. Color intensity was read at 734 nm by using an ultraviolet (UV)- spectrophotometer. The value of IC50 is determined by the sample concentration, which requires to scavenge 50% radical of ABTS. The below formula was applied to estimate the inhibition (%).

% Anti-radical activity = [(A0 - A1 / A0) x 100].

Where A0 - control of color intensity (blank, without extracts). A1 was the color intensity of the solvent extracts. The radical scavenging activity of vitamin C was also calculated and evaluated with the diverse solvent extracts.

### Radical scavenging method- DPPH:

The DPPH radical scavenging methodology was adopted according to the previous publications [42,43]. In vitro assay was achieved in microtitre plates (96 well). Added DPPH solution (200 µl) to different plant extracts (10 µl) or the standard solution and kept the plates at room temperature for 30 minutes incubation and the color intensity was read at 490 nm using a UV- spectrophotometer. The value of IC_50_ is determined by the sample concentration, which requires to scavenge 50% radical of DPPH.

% Anti-radical activity = [(A0 - A1 / A0) x 100]

Where A0 - control of color intensity (blank, without extracts). A1 was the color intensity of the solvent extracts.

### Radical inhibitory activity- NO:

NO radical inhibition assay was achieved based on the prior publications [44,45]. Added to sodium nitroprusside (4ml, 10 mM), PBS (1 ml), and plant extract in DMSO (1ml) and make up the final volume of 6 ml in the microtitre plates that were incubated at room temperature for 2 hours. Then, a suspension comprising nitrates (0.5 ml) was eliminated and added sulphanilic acid (1 ml), shaken well, and kept at the finishing point of diazotization for at least 5 min, and added NADD (1 ml), and kept in diffused light at 37°C for 30 min. The color intensity of the suspension was read at 540 mm using a UV- Spectrophotometer. The value of IC50 is determined by the sample concentration, requires inhibiting 50% NO radical.

### Radical scavenging method- HPO:

The scavenging of HPO radical was performed based on the former publications [45,46]. Added to different parts of the plant extracts (1 ml) in methanol to HPO (2 ml; 20 mM HPO in PBS). After 15 min incubation, the color intensity was read at 230 nm using a UV- Spectrophotometer. The blank was the plant suspension in PBS without HPO.

### Radical scavenging method- LPO:

LPO was assessed by the TBARS method in accordance with previous literature [47-49]. Added to 100 µl of the different plant extracts to lipid mixture (1 ml) and the blank was set without the extracts of the plant. The reaction of LPO was triggered by the addition of oxidant compounds, FeCl3 (400 mM, 10 µl) and ascorbic acid (200 mM, 20 µl), and kept incubation for an hour at 37°C. The reaction was cessation by the addition of HCl (0.25 N, 2 ml) containing TCA (15%) and TBA (0.375%) and the mixture was heated for 20 min; cooled; centrifuged, and then the color intensity of the suspension was read at 532 nm using UV-Spectrophotometer.

### Antimicrobial activity:

The antibacterial and antifungal activity was performed by the method of cup-plate [13,14,50]. Briefly, the sterile Petri plates were prepared using Sabourd dextrose agar or sterile nutrient agar (Himedia) and kept in aseptic conditions. About 100 µl of the test organisms [Gram-positive (Bacillus aerogenes, B. coagulans, B. megatarium, B. subtilis, Lactobacillus lichmani, Staphylococcus aureus), Gram-negative (Kleibsella pheumoniae, Pseudomonas aeruginosa, Salmonella typhi, Shigella), and fungal strains (Aspergillus niger, A. flavus, Candida albicans, Cryptococcus neoformans, Trichophyton rubrum)] were spread on the defined sterile plates. Holes (diameter, 5 mm) were made by a sterile bore in the plates. The standard antibiotics and plant extracts were poured into respective holes and the plates were kept for at least an hour at 4°C to permit the diffusion into the agar medium followed by 24-48 h incubation at 37°C.

### Assay of Minimum inhibitory concentration:

MIC was performed using different parts of the plant by the method of two-fold dilution [14,17,19,51-55]. Sequences of test tubes were used to contain an equal volume of medium spread with the test organisms. Reduced volume of test drugs was introduced into the tubes, typically, a succeeding two-fold dilution was adopted. In this, one tube remained without any test drug, aided as a positive control. The cultures were maintained in the incubator according to the respective strains and temperature (bacteria: 37°C for 24 hours, Fungi: 27°C for 48 hours) which is necessary for the multiplication of up to 15 generations of the strains. Then, the growth of the organisms was visualized based on the turbidity, however, antimicrobial agents in the tube inhibited the growth of the organism, showed transparency. Hence, MIC is the drug concentration existing in the clear tube, in which the lowest drug concentration could not generate any growth.

### Cytotoxic studies:

MTT assay:

In vitro cytotoxic investigation was achieved by MTT assay [56]. Briefly, the cultured cells were trypsinized and the viable cells (1.0 x 105 cells/ml) were inoculated in the medium containing 10 % BSA. About 0.1 ml of diluted cell suspension (10,000 cells) was added into each well of 96 well microtitre plates. A limited monolayer was formed after 24 hours and washed with the medium, added 100 µl of different drug concentrations. The plates were then kept in the CO_2_ incubator chamber at 37°C in a 5% CO_2_; microscopic observation was performed and noted every 24 hours. The drug in the well was washed and added 50 µl of MTT in DMEM medium without phenol red for 72 hours. Then the plates were gently mixed and kept in the CO_2_ incubator chamber for 3 hours. The upper suspension was removed and added propanol (50 µl). The color intensity was read at a wavelength of 540 nm using a microplate reader. The % of growth inhibition was measured by the following formula:

Growth inhibition (%) = 100− Mean OD of test Mean OD of Control X 100

### Assay of total cell protein content:

The assay of total cell protein composition was performed by SRB method [57]. The cultured cells were trypsinized and the viable cells (1.0 x 105 cells/ml) were inoculated in the medium containing 10 % BSA. About 0.1 ml of diluted cell suspension (10,000 cells) was added into each well of 96 well microtitre plates A limited monolayer was formed after 24 hours and washed with the medium, added 100 µl of drug concentrations. The plates were then kept in the CO_2_ incubator chamber at 37°C in a 5% CO_2_; microscopic observation was performed and noted each 24 h. Next 72 hours, 50% TCA (50 µl) was added to the wells and observed a tinny layer over the dilutions of the drug to become 10% concentrations; incubated for at least an hour at 4°C; then washed thrice to eliminate the turbid, and then air-dried. Then stained with SRB and maintained for 30 min under room temperature. The dye was then detached by quick washing using acetic acid (1%), air-dried, and mixed with 100 µl of Tris buffer (10 mM) to dissolve the dye. The color intensity was read at 540 nm using a microplate reader and calculated using the following calculation:

Growth inhibition (%) = 100− Mean OD of test Mean OD of Control X 100

### Studies of short-term toxicity:

A study of short-term toxicity and antitumor screening was achieved using DLA and EAC cells [58]. The cell suspension was inoculated into the mouse peritoneal cavity through injecting the dense cell volume of 1.0x 105 cells/ml. Then, the cells were introverted using a sterile syringe from the peritoneal cavity of the mouse while it became inflammation for about 12-14 days (This study was approved by the Committee on the Use of Live Animals in Teaching and Research of the university (Permit number: 4722-18). The cells were splashed thrice using HBBS and spinned for 3 min at 1,500 g. A known volume of HBSS used to suspend the cells and counting of the cells was maintained to 2 x 106 cells/ml. These cells were then treated with the drug and kept incubation for at least 3 hours. After incubation, a dye exclusion test was performed. The volume of the drug-treated cells and trypan blue (0.4%) equally were combined and loaded into a hemocytometer and the viable and nonviable cells were counted.

% Of inhibition = 100− Total Number of cells – Dead cells Total number of Cells X100

## Results:

Different plant parts (leaf, root, stem, and whole plant) used and the percentage of crude extracts of A. graveolens L. was presented in Table 1 (see PDF). All these extracts obtained by the cold maceration process, which were undergone to qualitative preliminary phytochemicals screening. The outcome of the study showed that A. graveolens L. contains the common secondary metabolites such as glycosides, tannins, saponins, flavonoids, steroids, terpenoids, alkaloids, carbohydrates, proteins, anthraquinones, are shown in Table 1 (see PDF). The whole plant of the A. graveolens L. showed higher antioxidant properties, listed in Table 2 (see PDF). The value of IC50 is determined by the sample concentration, requires inhibiting 50% NO radical. The leaf extract of A. graveolens L exposed the higher antioxidant properties with an IC50 value of 16.23 ± 0.147 µg/ml. In addition, the leaf extract of A. graveolens L. exhibited potent antioxidant activity in all DPPH, LPO, and hydrogen peroxide method when compared to the root, stem, and whole plant extracts. All four parts of A. graveolens L were screened for their antimicrobial effects against six Gram-positive viz., Bacillus aerogenes, B. coagulans, B. megatarium, B. subtilis, Lactobacillus lichmani, Staphylococcus aureus; four Gram-negative viz., Kleibsella pheumoniae, Pseudomonas aeruginosa, Salmonella typhi, Shigella strains and five fungal strains viz., Aspergillus niger, A. flavus, Candida albicans, Cryptococcus neoformans, Trichophyton rubrum which were performed by the cup-plate method; MIC was measured by the method of two-fold serial dilution, and both results are tabulated in Table 3 (see PDF). All four parts of A. graveolens L demonstrated to possess high inhibition against Gram-positive and negative strains extended between 12-21 mm and listed fungal strains had 07-21 mm. The value of MIC of A. graveolens L extended to 250-500 µg/ml and the outcomes are presented in Table 3 (see PDF). Cytotoxic investigations of A. graveolens L were achieved using five various cell lines namely, Vero, 3A, L-929, A-549, and L6.BRL is listed in Table 4 (see PDF). The cytotoxic analysis was determined using microbial growth inhibition, which was achieved by the common methods of MTT and SRB. The crude extracts of stem, leaf, root, and whole plant demonstrated moderate to high cytotoxic effects in all respective five-cell lines with the value of cytotoxicity-50 stretching between 443-168.5 µg/ml. Short-term toxicity was studied using DLA and EAC cells, listed in Table 5 (see PDF). The short-term antitumor investigation was done by DLA and EAC that were newly obtained from the mouse peritoneum. Both these cells were treated with the extract of the leaf with dilutions ranges from 62.5 µg/ml- 1000 µg/ml and viability of the cell was noted.

## Discussion:

The investigations for a selective and less noxious compound for bacterial infections and cancer treatment are an enduring progression. Plant-derived drugs have extensively been used in various nations for bacterial infections and cancers. WHO approves that plant-derived medicines aid the well-being essentials of around 75% of the global populace particularly people in rural areas in several developing nations. The current revival of herbal medicines might outcome from the efficiency and competence of various active compounds from the plants [59]. Most recent allopathy drugs have potential side effects and essential to be alternative to diminish these effects; plant-derived compounds are impressively promising without any side effects and readily accessible and easy to practice. Several pharma industries and research organizations are nowadays investigating the plant-derived compounds frequently due to their obtainability and medicinal values. For incidence, several investigators have been investigated on plants and their active principles that have served as a primary basis of effective antioxidant, anticancer, and antimicrobial agents. The outcomes of the studies strongly suggest that around 60% of the presently available anti-cancer and antimicrobial drugs are obtained from plant compounds [1,6,19,52,55]. The information of traditional medicinal plants to contemporary studies delivers a novel strategy that creates the rate of encounter of medications much faster than in random gatherings. In the present study, preliminary phytochemical screening of the A. graveolens showed the occurrence of glycosides, tannins, saponins, flavonoids, steroids, terpenoids, alkaloids, carbohydrates, proteins, and anthraquinones. Earlier, A. graveolens extract has been reported to contain luteolin-glycosides, β-pinene, β-phellendrene, terpinolene, camphene, γ-terpinene, cumene, limonene, α-pinene, p-cymene, sabinene, α-thuyene, and related furocoumarins, which have anticancer, antibacterial, and antifungal activities [60-63]. Antimicrobial activity of the crude extracts of A. graveolens has a noteworthy bactericidal activity against different Gram-positive, negative and fungal strains. It has been documented that the potency of plant extracts and their bioactive constituents are known to have antimicrobial activity [1,2,6,14,17,54, 64,65]. The highest phytochemicals especially flavonoids have been documented to have antibacterial activity [2]. The underlying mechanism of antibacterial action of flavonoids is recognized to DNA topoisomerase inhibition, plasma membrane degradation, and deteriorating of microbial energy metabolism [1,66,67]. Even though these phytochemical constituents are responsible for antibacterial and antifungal effects, it is suggested that they can be solely or in groupings with other metabolites to enable the plant as potentials [1]. ROS and RNS have manifold functions in the human body and are implicated in tumor initiation and progression [68]. Various studies showed that elevated these ROS and RNS and decreased endogenous antioxidant enzymes in the human tissues are well documented in carcinogenesis [69,70]. Many tumor cells in the human body have been recognized as pro-oxidant and augment inflammatory reactions and oxidative stress. Elevated these oxidative stress upsurges the enduring capacity of the tumor cells by elevation and activation of mutations, signaling of redox reactions, and proinflammatory factors, chemokines, NF-kB, and cytokines [71-74]. Antioxidants modify this intracellular redox status and thus elevating the effects of cytotoxic therapy. In the present study, different parts of the A. graveolens extract significantly scavenged NO, DPPH, ABTS, Lipid peroxidation, and hydrogen peroxide, which show its potential antioxidant activity. Earlier, It was documented that extracts of the plant containing antioxidant potential demonstrated effective cytotoxicity toward various cancer cell lines [69] as well as antineoplastic effects in various animal models [18]. The underlying mechanisms of cytotoxicity and anticancer activity of the compounds obtained from plants are often the activation of apoptosis, inhibition of pro-inflammatory signaling, and or inhibition of angiogenesis [63]. Plants with secondary metabolites especially high contents of phenolic acid and flavonoids demonstrate to have potential antioxidant and anticancer activities [7, 75] and different parts of crude extracts of A. graveolens have been identified to have high contents of phenolic acid and flavonoid from the antioxidant investigations made, therefore might have anticancer properties. The study of short term in vitro cytotoxic numbers also shows that anticancer activity of crude extracts of A. graveolens presented counter to EAC and DLA, thereby A. graveolens caused noteworthy cancer cell demise and cell growth inhibition in all respective six different cancer cell lines. Based on the present study, the constituents of any of the metabolites in the plants, especially, flavonoids and phenolic acid constituents may be recognized to the anticancer activity of the plant extract. The current study focuses on the preliminary phytochemical screening, antioxidant, and anticancer activity of A. graveolens; the underlying mechanisms of cancer-selective mechanisms and the active principles of A. graveolens accountable for the activity are underway.

## Conclusion

Data shows that crude extract of A. graveolens exhibit cytotoxic, antibacterial, antifungal, and antioxidant activity. The plant extract contains bioactive principles that are known to be free radical scavengers, cytotoxic against various tumor cell lines, and active in the inhibition of Gram-positive, Gram-negative, and fungal strains for further consideration.
